# Oncogenic IDH1 Mutations Promote Enhanced Proline Synthesis through PYCR1 to Support the Maintenance of Mitochondrial Redox Homeostasis

**DOI:** 10.1016/j.celrep.2018.02.084

**Published:** 2018-03-20

**Authors:** Kate E.R. Hollinshead, Haydn Munford, Katherine L. Eales, Chiara Bardella, Chunjie Li, Cristina Escribano-Gonzalez, Alpesh Thakker, Yannic Nonnenmacher, Katarina Kluckova, Mark Jeeves, Robert Murren, Federica Cuozzo, Dan Ye, Giulio Laurenti, Wei Zhu, Karsten Hiller, David J. Hodson, Wei Hua, Ian P. Tomlinson, Christian Ludwig, Ying Mao, Daniel A. Tennant

**Affiliations:** 1 Institute of Metabolism and Systems Research, University of Birmingham, Edgbaston, Birmingham B15 2TT, UK; 2 Institute of Cancer and Genomic Sciences, University of Birmingham, Edgbaston, Birmingham B15 2TT, UK; 3 Molecular & Population Genetics Laboratory, Wellcome Trust Centre for Human Genetics, University of Oxford, Oxford OX3 7BN, UK; 4 Department of Neurosurgery, Huashan Hospital, Fudan University, #12 Middle Wulumuqi Road, Shanghai 200040, China; 5 Institute of Biomedical Sciences, Fudan University, #131 Dong’an Road, Shanghai 200040, China; 6 Department of Bioinformatics and Biochemistry, Technische Universität Braunschweig, 38106 Braunschweig, Germany; 7 Centre of Membrane Proteins and Receptors (COMPARE), University of Birmingham and University of Nottingham, Midlands, UK; 8 State Key Laboratory of Medical Neurobiology, School of Basic Medical Sciences and Institutes of Brain Science, Fudan University, Shanghai 200040, China; 9 The Collaborative Innovation Center for Brain Science, Fudan University, Shanghai, 200040, China

**Keywords:** glioma, IDH1, redox, metabolism, proline

## Abstract

Since the discovery of mutations in isocitrate dehydrogenase 1 (IDH1) in gliomas and other tumors, significant efforts have been made to gain a deeper understanding of the consequences of this oncogenic mutation. One aspect of the neomorphic function of the IDH1 R132H enzyme that has received less attention is the perturbation of cellular redox homeostasis. Here, we describe a biosynthetic pathway exhibited by cells expressing mutant IDH1. By virtue of a change in cellular redox homeostasis, IDH1-mutated cells synthesize excess glutamine-derived proline through enhanced activity of pyrroline 5-carboxylate reductase 1 (PYCR1), coupled to NADH oxidation. Enhanced proline biosynthesis partially uncouples the electron transport chain from tricarboxylic acid (TCA) cycle activity through the maintenance of a lower NADH/NAD^+^ ratio and subsequent reduction in oxygen consumption. Thus, we have uncovered a mechanism by which tumor cell survival may be promoted in conditions associated with perturbed redox homeostasis, as occurs in IDH1-mutated glioma.

## Highlights

• IDH1 mutant cells exhibit increased PYCR1-dependent proline synthesis from glutamine
• The increase in proline synthesis is coupled to the oxidation of mitochondrial NADH
• PYCR1 activity partially uncouples respiration from TCA cycle activity
• PYCR1 expression and proline concentrations are increased in IDH1-mutated gliomas

## Graphical Abstract

**Figure:**
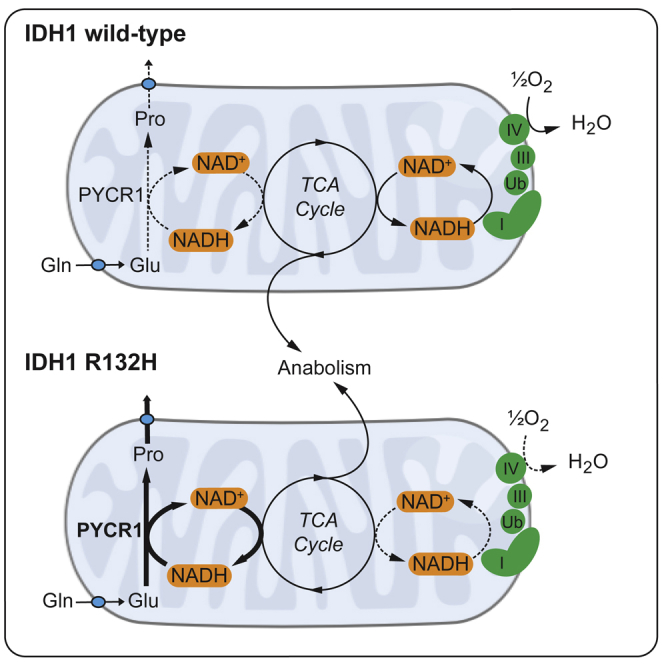

## Teaser

Hollinshead et al. demonstrate a role for PYCR1 in control of mitochondrial redox homeostasis. Expression of IDH1 R132H mutation leads to increased NADH-coupled proline biosynthesis, mediated by PYCR1. The resulting metabolic phenotype partially uncouples mitochondrial NADH oxidation from respiration, representing an oxygen-sparing metabolic phenotype.

## Introduction

Mitochondria constitute the major metabolic hubs of the eukaryotic cell, coordinating the metabolism of different nutrients to provide the macromolecular building blocks and energy required for cell function. Cell phenotype is, therefore, greatly dependent on appropriate mitochondrial metabolic activity (Chandel, 2015), and dysfunction could contribute to or drive disease. Indeed, it was postulated by Otto Warburg in the early 20^th^ century that mitochondrial metabolic dysfunction is the origin of cellular transformation (Warburg, 1956). Although this is now considered not to be a universal paradigm, the first genetic evidence to support this concept was uncovered at the turn of the 21^st^ century, where mutations in members of the mitochondrial succinate dehydrogenase (SDH) complex were discovered to be founder lesions in hereditary paragangliomas (Baysal et al., 2000). Since then, mutations in three mitochondrial enzymes and one mitochondrial-associated enzyme have been shown to drive tumorigenesis; SDHA-D, fumarate hydratase (FH), and isocitrate dehydrogenases 1 and 2 (IDH1 and IDH2, respectively) (Frezza et al., 2011a).

Mutations in IDH1 are observed in a number of tumor types, including the majority of low-grade gliomas and secondary glioblastomas. The mutation observed is a heterozygous missense mutation in the codon for arginine 132 (R132), most commonly to histidine in gliomas R132H, although a number of other rare substitutions are also found (Yan et al., 2009). This mutation has been shown to result in a loss and gain of function: while NADP^+^-linked oxidation of isocitrate is lost, NADPH-coupled reduction of α-ketoglutarate is gained, with the resulting production of (R)-2-hydroxyglutarate (2HG) (Dang et al., 2009). Some of the major effects of the often millimolar intracellular concentrations of 2HG on cell phenotype are now known, including epigenetic changes and alterations in the cellular response to hypoxia (Losman and Kaelin, 2013).

One aspect of mutant IDH1 biology that has received less attention is the potential effect of its oncogenic function on cellular redox homeostasis. Cells expressing mutant IDH1 both lose a source of NADPH-reducing equivalents and acquire novel NADPH-coupled α-ketoglutarate-reducing activity (Lewis et al., 2014). This results in a change in the cellular NADPH:NADP^+^ ratio that manifests as an altered glutathione (GSH:GSSG) ratio (Bisdas et al., 2016), as well as sensitization to oxidative stimuli (Mohrenz et al., 2013, Shi et al., 2015). However, reducing equivalents are also transferred between the cytosolic and mitochondrial pyridine pools, through the use of direct translocation (e.g., nicotinamide nucleotide translocator; NNT), or through metabolic cycles such as the malate-aspartate or isocitrate-α-ketoglutarate shuttles. As such, any perturbation in redox homeostasis elicited by mutations in IDH1 may not be confined to the cytosol but have implications for tumor growth through altered regulation of mitochondrial metabolism (Grassian et al., 2014).

Therefore, we sought to investigate whether the mitochondria of IDH1 R132H-expressing cells compensate for the altered cytosolic redox state through changes in redox-active metabolic pathways. We found that IDH1 mutant cells exhibit increased NADH-coupled pyrroline 5-carboxylate reductase 1 (PYCR1)-dependent proline synthesis from glutamine, which resulted in the partial uncoupling of respiration from tricarboxylic acid (TCA) cycle activity. Furthermore, we found that IDH1 mutant gliomas exhibit increased PYCR1 expression and that tumoral 2HG concentrations correlated with that of proline, suggesting that this effect is also observed in glioma patients.

## Results

### IDH1 Mutation Induces Glutamine-Derived Proline Synthesis

**Figure 1:**
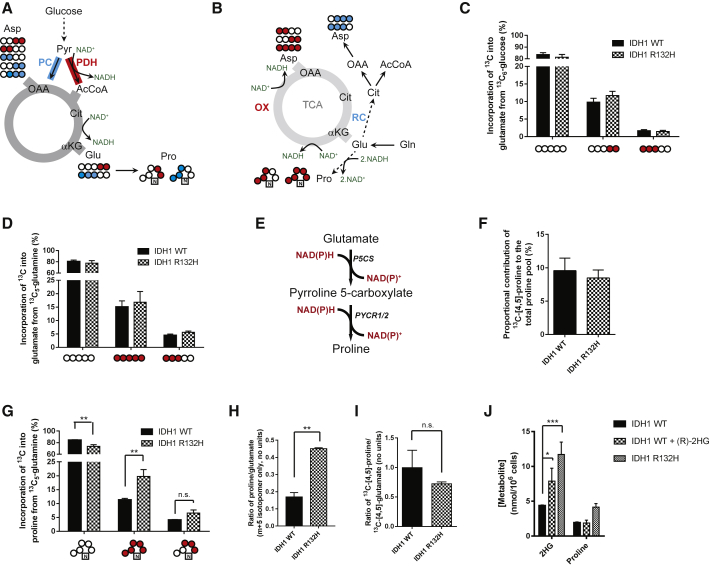
Increased Proline Synthesis in IDH1 Mutant Cells (A) Alternative entry points of pyruvate into the TCA cycle and the implications for ^13^C incorporation into metabolites from ^13^C_6_-glucose. (B) Incorporation of ^13^C_5_-glutamine into the TCA cycle. (C) Incorporation of glucose-derived pyruvate into glutamate shows no difference between WT and IDH1 R132H-expressing cells. (D) ^13^C incorporation into glutamate from ^13^C_5_-glutamine also shows no significant differences between IDH1 mutant and WT cells. (E) Diagram showing the synthesis of proline from glutamate, with 2 mol NAD(P)H oxidized per mole of glutamate to proline. (F) The contribution of glucose carbons to the proline pool is unchanged in cells expressing mutant IDH1. (G) Glutamine carbons that arise directly from glutamate (without a pass through the TCA cycle) are significantly increased as a proportion of the proline pool. Absolute values were calculated from ^1^H-NMR spectra. (H and I) In (H), when the proline synthesized from glutamine is normalized to take into account relative changes in glutamate labeling (m + 5 proline/m + 5 glutamate; gas chromatography-mass spectrometry [GC-MS]), this significance is retained, while (I) ^13^C-[4,5]-proline arising from ^13^C-[4,5]-glucose remains unchanged (HSQC NMR spectra). (J) IDH1 WT cells incubated with 10 mM (R)-2HG elicits an intermediate 2HG intracellular concentration that does not increase proline synthesis (calculated from ^1^H NMR spectra). All error bars represent mean ± SEM. Statistical tests used for both (G) and (I): 2-way ANOVA with post hoc test to identify individual significant changes. In (H) and (I), comparisons were performed using a Mann-Whitney test. ^∗^p < 0.05; ^∗∗^p < 0.01; ^∗∗∗^p < 0.001; n.s., not significant. αKG, α-ketoglutarate; AcCoA, acetyl coenzyme A; Asp, aspartate; Cit, citrate; Glu, glutamate; OAA, oxaloacetate; OX, oxidative TCA cycle; PC, pyruvate carboxylase; PDH, pyruvate dehydrogenase; Pro, proline; RC, reductive carboxylation; TCA, tricarboxylic acid.

To investigate the changes in redox-active pathways induced by the IDH1 R132H mutation, we utilized a human anaplastic oligodendroglioma (HOG) cell line engineered to express either wild-type (WT) or mutant (R132H) IDH1 (Reitman et al., 2011) (Figure S1A). ^13^C_6_-glucose or ^13^C_5_-glutamine was used as the carbon source to investigate the effects of mutant IDH1 expression on mitochondrial redox-dependent metabolism (Figures 1A and 1B). The synthesis of glutamate from glucose, which occurs through the metabolism of pyruvate in mitochondria, can be either through NAD^+^-linked pyruvate dehydrogenase (PDH) activity or through pyruvate carboxylase (PC), which is redox neutral. These two activities can be differentially observed though the production of two isotopomers of glutamate: ^13^C-[4,5]-glutamate (for PDH) and ^13^C-[1,2,3]-glutamate (for PC; Figure 1A). We observed that the distribution of isotopomers was similar between IDH1 WT and mutant cells, suggesting that there was little detectable alteration in overall NADH:NAD^+^ regulation through these pathways (Figure 1C). Similarly, the synthesis of glutamate from glutamine, a single deamidation step, was also unchanged (Figure 1D). Therefore, we analyzed the incorporation of ^13^C from both carbon sources into aspartate, which includes further redox-active enzymatic steps (α-ketoglutarate and malate dehydrogenases). Again, both the glucose-derived oxidative isotopomers of aspartate (^13^C-[1,2]/[3,4]-aspartate) as well as the glutamine-derived ^13^C_4_-aspartate were unchanged (Figures S1B and S1C).

An alternative, redox-active mitochondrial metabolic pathway is the synthesis of proline from glutamate, which oxidizes 2 mol of NAD(P)H per mole of proline synthesized (Figure 1E). This has previously been suggested to play a role in the cellular redox stress response (Krishnan et al., 2008, Lorans and Phang, 1981). Although no change in ^13^C enrichment into proline was observed from glucose (Figure 1F), a significantly larger proportion of the proline pool was enriched from glutamine in mutant IDH1 cells (Figure 1G). As the ^13^C_5_ isotopomer of proline is synthesized directly from the ^13^C_5_-glutamate isotopomer, the ratio of the two provides an indication of the contribution of glutamine-derived glutamate to the proline pool. This was significantly increased in IDH1 mutant cells compared to IDH1 WT (Figure 1H). Notably, this was different from the synthesis of glutamate-derived proline produced from glucose (Figure 1I), despite both glutamate pools being within the mitochondrial matrix. These data, therefore, suggest that the IDH1 mutation elicits increased proline synthesis selectively from glutamine. To determine whether IDH2 mutant cell lines would also demonstrate a similar response, we created two cell lines using the WT IDH LN18 glioma as parental, to either express IDH1 R132H or IDH2 R172K (Figures S1D and S1E). We found that, although the IDH1 R132H-expressing cell line recapitulated the proline synthetic phenotype observed in the HOG line, the IDH2 R172K cell line did not (Figures S1F and S1G). This suggests that the metabolic perturbation is specific to the IDH1 isozyme. Many of the phenotypes reported in IDH1-mutated cells, including the induction of cellular transformation, have been shown to be secondary to the production of 2HG (Losman et al., 2013, Lu et al., 2012). However, our data using the IDH2 R172K cell line, which also synthesizes significant 2HG compared to IDH1 R132H-expressing lines (Figures S1H and S1I), suggested that the proline synthetic phenotype may be independent of this oncometabolite. Therefore, we tested whether the observed change in proline metabolism was, indeed, 2HG independent by incubating IDH1 WT cells with 10 mM (R)-2HG for 48 hr prior to metabolite extraction. 2HG incubation produced intracellular 2HG concentrations that were intermediate between WT and mutant IDH1-expressing cells (Figure 1J). However, no increase in intracellular proline was observed in these conditions, suggesting that the phenotype was unlikely to result from increased intracellular 2HG but, rather, the metabolic consequences arising from the expression of mutant IDH1.

**Figure 2:**
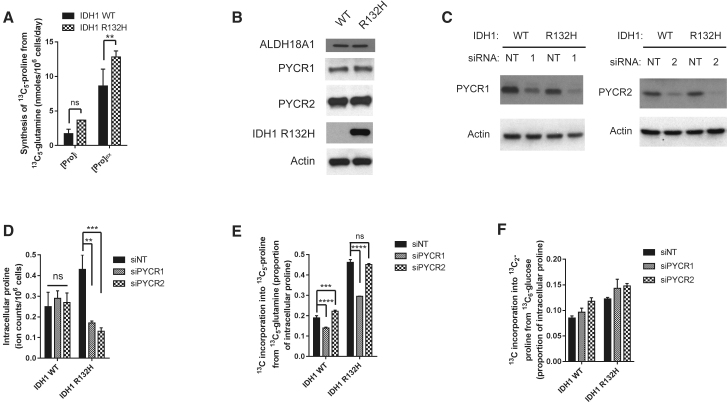
Enhanced Proline Synthesis Is through Increased PYCR1 Activity in IDH1 Mutant Cells (A) The absolute amount of proline synthesized from glutamine (calculated from ^1^H-NMR spectra) is significantly increased in the medium. (B) IDH1 mutant cells do not increase proline synthesis through upregulation of the mitochondrial proline biosynthetic enzymes. (C) Confirmation of knockdown of PYCR1 or PYCR2 in both IDH1 WT and mutant cells. (D) Intracellular proline is unchanged in IDH1 WT cells after siPYCR1 or siPYCR2 but significantly reduced in IDH1 mutant cells in response to either PYCR1 or PYCR2 (ion counts per 10^6^ cells). (E) Incorporation of ^13^C from glutamine into proline is significantly decreased after siPYCR1 in IDH1 mutant cells (∼40%) compared to (∼25%) in IDH1 WT cells. (F) Neither siPYCR1 or siPYCR2 decreased incorporation of glucose into proline. All error bars represent mean ± SEM. A 2-way ANOVA with a post hoc test was used for both (A) and (E). ^∗∗^p < 0.01; ^∗∗∗^p < 0.001; ^∗∗∗∗^p < 0.0001; ns, not significant.

It has previously been shown that proline catabolism, which is also redox active, plays a role in malignant cancer cell phenotype (Elia et al., 2017). The enhanced synthesis of proline observed here could, therefore, either be part of a cycle to shuttle reducing equivalents between the cytosol and the mitochondria (Hagedorn and Phang, 1983) or a means of oxidizing NADH to bypass the electron transport chain (ETC). If the latter, then the proline synthesized must be removed from the cell as mitochondrial PYCR enzymes are significantly product inhibited (De Ingeniis et al., 2012). Therefore, we compared the concentrations of ^13^C_5_-proline in cell extracts and the media. Although intracellular concentrations of ^13^C_5_-proline were not appreciably different, extracellular ^13^C_5_-proline concentrations were significantly higher in the medium from IDH1 mutant cells compared to WT, consistent with increased synthesis of proline that was not balanced by catabolism (Figure 2A). This was observed even in the presence of concentrations of exogenous proline found in the peripheral plasma (Figure S1J).

### Enhanced Proline Synthesis in IDH1-Mutated Cells Is Mediated through PYCR1

We therefore examined whether IDH1 mutant cells demonstrated altered proline biosynthetic enzyme expression. Proline is synthesized from glutamate in two steps; pyrroline 5-carboxylate (P5C) synthase (encoded by *ALDH18A1*) followed by either of two P5C reductases (PYCR1 and PYCR2; Figure 1E). The expression of all three enzymes was found to be similar between IDH1 WT and R132H-expressing cell lines at both protein (Figures 2B and S2A) and mRNA (Figure S2B) levels, further suggesting that the altered synthesis of proline observed in IDH1 mutant cells was through a change in redox regulation. To investigate the role of PYCR1 and PYCR2 on proline metabolism, we induced transient knockdown of each PYCR isozyme (Figures 2C and S2C) and assessed the effect on proline synthesis from glutamine. Although little decrease in the intracellular steady-state concentration of proline was found in the IDH1 WT cells with either siPYCR1 or siPYCR2 (Figure 2D), a significant decrease occurred in IDH1 R132H-expressing cells with both siPYCR1 and siPYCR2. The effect of PYCR1 was confirmed in the LN18 cell model (Figure S2D). These data suggest that the PYCR1 and PYCR2 are the major proline synthetic enzymes in IDH1 R132H mutant cells.

Since proline synthesized from glucose was not increased in IDH1 mutant-expressing cells (Figures 1F–1I), we wondered whether PYCR1 and PYCR2 are responsible for synthesizing proline from different carbon sources. We first assessed incorporation of ^13^C from glutamine into proline in the presence of siPYCR1 or siPYCR2 and found that siPYCR1 resulted in a reduction in ^13^C_5_-proline (Figures 2E and S2E). In contrast, reduction of neither PYCR isozymes affected proline synthesis from glucose (Figure 2F), providing evidence for a selective use of glutamine-derived glutamate as a source of proline in IDH1 R132H-expressing cells.

### Increased PYCR1 Expression and Proline Synthesis Is Observed in IDH1-Mutated Gliomas

**Figure 3:**
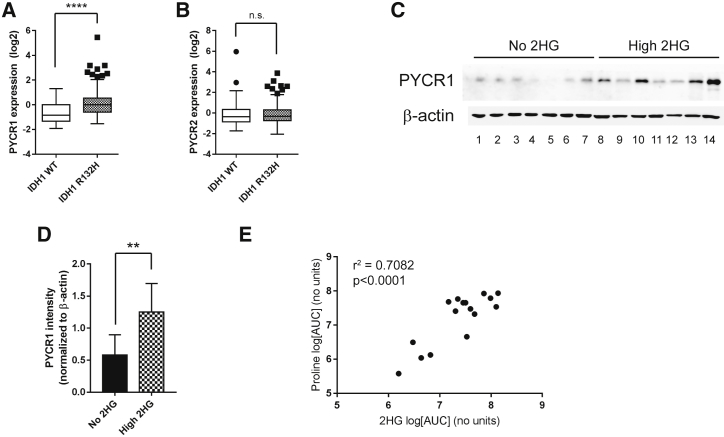
PYCR1 Expression and Proline Synthesis Is Correlated with Mutant IDH1 Activity in Gliomas (A and B) The results shown here are based in whole or partly upon data generated by The Cancer Genome Atlas (TCGA) Research Network (http://cancergenome.nih.gov/). PYCR1 (A) and PYCR2 (B) gene expression data from 60 IDH1 WT and 221 IDH1 R132X low-grade gliomas show that PYCR1 is significantly increased in IDH1 mutant gliomas. (C) Immunoblotting for PYCR1 in gliomas suggest that expression is increased with 2HG levels. Patient information for each lane is shown in Table S1. (D) Quantification of immunoblot in (C) showing a significant upregulation of expression of PYCR1. (E) Proline concentrations in gliomas correlate with 2HG concentration with an r^2^ value of 0.7082. Pearson r value: 0.842, p < 0.0001. Patient information for this cohort is shown in Table S2. All error bars represent mean ± SEM. The Mann-Whitney test was used for statistical tests performed in (A) and (D). ^∗∗^p < 0.01; ^∗∗∗∗^p < 0.0001; n.s., not significant. See also Tables S1 and S2.

It is apparent from studies using IDH1 R132H-expressing cells, including the above HOG cell model, that although expression of the mutant enzyme may be stable in culture (Figure S1A), 2HG steady-state concentrations may alter over time (Figure 1J; Figure S1K) (Reitman et al., 2011). To determine whether the same phenotype was observable *in viv*o, we first investigated the expression of PYCR1 and PYCR2 in low-grade gliomas, in which IDH1 mutations are commonly observed (Yan et al., 2009). Expression data from 285 lower grade gliomas available through The Cancer Genome Atlas (Cerami et al., 2012, Gao et al., 2013) were analyzed. We found that expression of PYCR1, but not PYCR2, was increased in IDH1-mutated tumors (Figures 3A and 3B). We further investigated PYCR1 expression in IDH1 WT and mutated gliomas and confirmed that tumors with high 2HG concentrations (Figures S3A and S3B, for examples) also demonstrated increased expression of PYCR1 (Figures 3C, 3D, and S3C–S3F). Based on this, we directly measured proline and 2HG concentrations in IDH1-mutated gliomas and found that the steady-state levels of these metabolites were significantly correlated (Figure 3E). This observation adds proline to a growing list of amino acids, the metabolism of which is altered in tumors expressing the IDH1 mutation. However, collectively, the *in vitro* and *in vivo* data, therefore, suggest that PYCR1 may play a role in redox regulation in IDH1-mutated gliomas.

### Mitochondrial NADH-Oxidizing Activity of PYCR1 Is Oxygen Sparing

**Figure 4:**
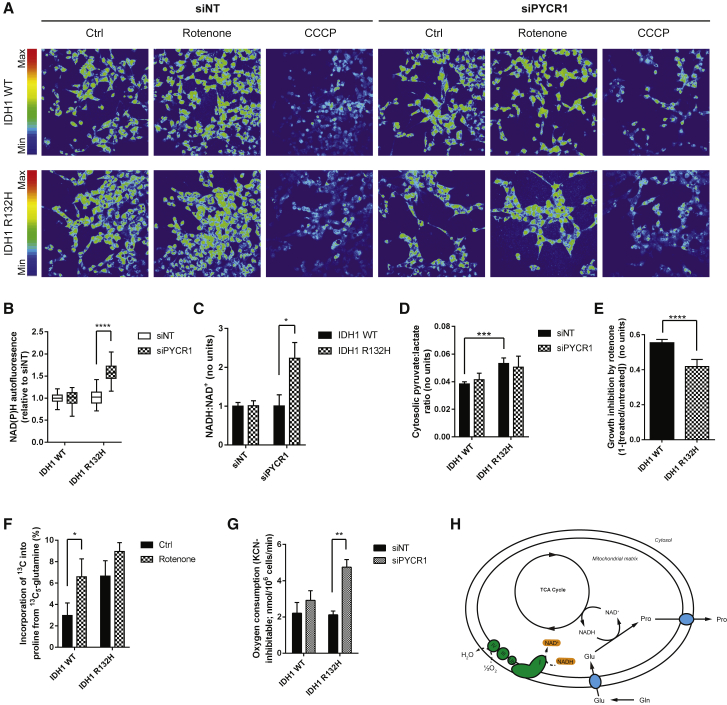
PYCR1 Activity in IDH1 Mutant Cells Oxidizes Mitochondrial NADH (A) NAD(P)H autofluorescence shows that knockdown of PYCR1 results in increased NAD(P)H autofluorescence only in IDH1 mutant cells. (B) Quantification of (A). (C) Biochemical quantification of the NADH:NAD^+^ ratio confirms that PYCR1 activity oxidizes NADH in IDH1 mutant cells. (D) The pyruvate:lactate ratio in IDH1 WT and mutant cells quantified using GC-MS data shows that, although the ratio is larger in IDH1 mutant cells, siPYCR1 does not alter it. (E) Sub-maximal concentrations of rotenone inhibit the proliferation of IDH1 WT cells significantly more than IDH1 mutant cells. (F) Inhibition of complex I results in a significant compensatory increase in glutamine-derived proline synthesis in IDH1 WT cells to a level similar to that of IDH1 mutants. (G) Knockdown of PYCR1 results in increased respiration only in IDH1 mutant cells. (H) Schematic showing the mechanism by which enhanced PYCR1 activity permits increased anabolism in IDH1 mutant cells. All error bars represent mean ± SEM. Statistical analyses where shown: 2-way ANOVA with a post hoc test of the relevant pairs of samples. ^∗^p < 0.05; ^∗∗^p < 0.01; ^∗∗∗^p < 0.001; ^∗∗∗∗^p < 0.0001.

PYCR1 has been reported to have a significantly higher affinity for NADH compared to NADPH (De Ingeniis et al., 2012), the former being the major pyridine species within most mammalian cells. Therefore, we examined the effect of siPYCR1 on overall cellular redox homeostasis using the autofluorescence of reduced pyridine nucleotides (Frezza et al., 2011b). While siPYCR1 had no effect on relative pyridine autofluorescence in IDH1 WT cells, it resulted in a significant increase in IDH1 mutant cells (Figures 4A and 4B), suggesting that PYCR1 can play a significant role in modulating cellular redox. These data were supported by direct measurement of the NADH:NAD^+^ ratio (Figures 4C, S4A, and S4B), confirming that PYCR1 plays a significant role in determining the NAD^+^:NADH ratio in IDH1 mutant cells. To assess whether the altered NADH:NAD^+^ ratio was a result of cytosolic or mitochondrial metabolism, the pyruvate:lactate ratio was assessed as a cytosolic surrogate for NADH:NAD^+^. Although the ratio in IDH1 R132H-expressing cells was significantly shifted toward NAD^+^ (i.e., increased pyruvate:lactate ratio), no change was observed in either background following PYCR1 knockdown (Figures 4D and S4C). To investigate whether this alteration in cellular redox homeostasis affects the antioxidant capacity or oxidative stress in IDH1 mutant cells, we examined the GSH:GSSG ratio—a readout of the major antioxidant response redox couple—and reactive oxygen species levels. In both HOG and LN18 cell models, siPYCR1 resulted in no significant change to either readout (Figures S4D and S4E).

These results suggest that PYCR1 activity in IDH1 mutant cells supports mitochondrial NADH oxidation. Complex I is the major site of NADH oxidation linked to oxygen consumption and proton pumping for ATP generation. Inhibition of complex I activity would be expected to limit proliferation through the restriction of oxidative TCA cycle activity. Although this was the case in the IDH1 WT cells, the NADH-oxidizing activity of PYCR1 appeared to act as a “metabolic bypass” of complex I, permitting continued proliferation in the presence of rotenone (Figure 4E). To investigate whether this bypass could be induced in IDH1 WT cells by increasing the mitochondrial NADH:NAD^+^ ratio, incorporation of ^13^C_5_-glutamine into proline was assessed in the presence and absence of rotenone. IDH1 WT cells treated with rotenone demonstrated a significant compensatory increase in proline synthesis, with IDH1 mutant cells showing a smaller increase (Figure 4F). This supports the notion that PYCR1 activity can be induced by increased mitochondrial NADH:NAD^+^ ratios, thereby retaining TCA cycle activity when ETC flux is limiting. These data also suggest that inhibition of PYCR1 activity may induce a compensatory increase in ETC activity to maintain cellular anabolism. Respiration in IDH1 WT and R132H-expressing cells was, therefore, investigated after siPYCR1. Although little effect was noted in IDH1 WT cells, siPYCR1 significantly increased oxygen consumption in IDH1 R132H cells (Figures 4G and S4F), confirming this compensatory mechanism.

The enhanced PYCR1 activity in IDH1 mutant cells would be expected to support the previously reported finding of continued oxidative TCA cycle function in limiting oxygen tensions (Grassian et al., 2014), which we also note in this cell model, using oxidative synthesis of aspartate as a readout (Figure S4G) (Birsoy et al., 2015, Sullivan et al., 2015). Our data further imply that increased PYCR1 activity in IDH1 R132H-mutated cells is likely to support greater cell proliferation for a given respiratory activity. This may play a particularly important role in gliomas, which are highly hypoxic, thereby limiting oxidative TCA cycle activity.

## Discussion

Since the discovery of mutations in IDH1 in gliomas and other tumors, significant strides have been made to better understand the downstream consequences of this oncogenic mutation. However, one aspect of the neomorphic function of the IDH1 R132H enzyme that has not garnered enough attention is the perturbation of cellular redox homeostasis that it elicits.

Through comprehensive investigation of the regulation of central carbon metabolism, we are able to show that glioma cells expressing mutant IDH1 increase the synthesis of proline through the activity of PYCR1, a mitochondrial NADH-oxidising enzyme. Our data suggest that, through the maintenance of a lower NADH:NAD^+^ ratio, the redox activity of PYCR1 partially uncouples the TCA cycle from respiration, permitting oxygen-independent synthesis of anabolic precursors, such as aspartate and citrate. This is an important finding, as it has been recently suggested that a major role of respiration in proliferating cells, in addition to the production of ATP, is to provide electron acceptors for the synthesis of aspartate (Birsoy et al., 2015, Sullivan et al., 2015). Indeed, this is likely to become increasingly important in hypoxic conditions, such as those observed in the tumor microenvironment, and may explain why oxidative TCA metabolism is increased in IDH1 mutant cells, even when oxygen becomes limiting (Grassian et al., 2014). It is important to note that the 2HG produced by mutant IDH1 may interfere with cell survival and proliferation in hypoxia by inhibiting the stabilization of the transcription factor, hypoxia inducible factor 1 (HIF1) (Koivunen et al., 2012, Tarhonskaya et al., 2014). It is, therefore, not clear what the overall effect of the IDH1 mutation is on hypoxic tumor phenotype.

The metabolism of the major carbon sources in cancer cells is not only used for cellular anabolism but also to maintain redox homeostasis—a particularly well-characterized cytosolic example of this being the NADH-coupled reduction of pyruvate to lactate by lactate dehydrogenase to sustain glycolysis. The data presented here suggest that the reduction of glutamate to proline through PYCR1 may be used as a means of maintaining mitochondrial redox homeostasis in hostile environments, such as oxidative stress and limiting oxygen tensions. Consistent with this, upregulation of PYCR1 has previously been suggested to form part of a metabolic transcriptional response to hypoxia in a number of tumors (Haider et al., 2016). Indeed, proline metabolism may be frequently dysregulated in cancers, with evidence suggesting increased synthesis (Filipp et al., 2012, Jain et al., 2012) and, conversely, catabolism (Elia et al., 2017). It will be important to deconvolute the regulation of proline activity at different points of the malignant progression of tumors if novel therapies are to be designed to perturb this aspect of metabolism.

In summary, we describe a stress-responsive metabolic pathway characterized by the synthesis of proline through PYCR1 to sustain cellular anabolism while sparing oxygen. This finding is likely to be an important metabolic bypass more generally, which could be hypothesized to permit enhanced tumor cell viability in hostile conditions, thereby contributing to the malignant progression of tumors.

## Experimental Procedures

### Cell Culture

A previously characterized human anaplastic oligodendroglioma (HOG) was used for all studies, unless otherwise stated, kindly donated by Professor Hai Yan (Reitman et al., 2011). The LN18 IDH1 WT, IDH1 R132H-expressing, and IDH2 R172K-expressing cell lines were made as described in the Supplemental Experimental Procedures. Cells were maintained in high-glucose DMEM supplemented with 10% FBS (Thermo Fisher Scientific, UK) and 2 mM L-glutamine in standard conditions. Knockdown of PYCR1 and PYCR2 was achieved using ON-TARGETplus pools. Tracing experiments were performed for 24 hr in basic formulation DMEM supplemented with either 10 mM ^13^C_6_ glucose or 2 mM ^13^C_5_ glutamine.

### Tumor Collection and Analysis

Tumors were collected under an approved institutional study (Huashan Hospital Ethics Committee, Shanghai, P.R. China), and informed consent was obtained from each patient under institutional review board protocols. Cohort information is shown in Tables S1 and S2. Metabolite analyses were performed as described in the Supplemental Information.

### Immunoblotting

Cells were lysed directly into Laemmli buffer and run on denaturing reducing PAGE before being transferred onto nitrocellulose membrane (GE Healthcare, UK). After blocking, membranes were incubated with one of the following antibodies: β-actin (Sigma, A4700, 1:4,000), PYCR1, and PYCR2 (Proteintech, 13108-1-AP, 1:5,000 and 17146-1-AP, 1:1,000, respectively). After washing and incubation with appropriate horseradish peroxidase (HRP)-linked secondary antibody (Cell Signaling), membranes were developed using EZ-ECL (Biological Industries, UK).

### NMR Spectroscopy

Cells washed with ice-cold 0.9% saline solution were extracted in 1:1:1 methanol, water, and chloroform. After shaking and centrifugation, the upper aqueous phase was collected and dried under vacuum. Samples were re-suspended in 100 mM sodium phosphate buffer (pH 7.0) containing 500 μM 2,2-dimethyl-2-silapentane-5-sulfonate (DSS) and 2 mM imidazole and 10% D_2_O into 1.7-mm nuclear magnetic resonance (NMR) tubes. 1D ^1^H-NMR spectra and 2D ^1^H,^13^C-heteronuclear single quantum coherence (HSQC) spectroscopy NMR spectra were acquired and analyzed as detailed in the Supplemental Information.

### Gas Chromatography-Mass Spectrometry

Cells washed with ice-cold 0.9% saline solution were extracted in 1:1:1 pre-chilled methanol, HPLC-grade water containing 1 μg/mL D6-glutaric acid (C/D/N isotopes) and chloroform. After shaking and centrifugation, the upper aqueous phase was collected and evaporated in gas chromatography (GC) glass vials under vacuum. Details on polar metabolite derivatization, data acquisition, and analysis can be found in the Supplemental Information.

### Redox Measurements

NAD(P)H was excited at λ = 351/364 nm, and autofluorescence was captured at λ = 385–470 nm. Carbonyl cyanine m-chlorophenyl hydrazine (CCCP; 20 μM) and rotenone (60 μM) were added to each well to achieve basal and maximal NAD(P)H autofluorescence, respectively. An NAD:NADH assay (NAD/NADH Glo Assay; Promega, G9071) was performed as per the manufacturer’s protocol.

### O_2_ Consumption Measurements

Oxygen consumption measurements were made using a Clark-type oxygen electrode (Oxytherm, Hansatech Instruments, Norfolk, UK). For details, see the Supplemental Information.

### Statistical Analysis

Samples sizes, reproducibility, and statistical tests used to analyze the datasets are described in the figure legends. Briefly, Mann-Whitney (2 samples) or 2-way ANOVA (≥2 sample groups) was used as appropriate, with multiple-comparisons post hoc tests as required. Unless otherwise noted, all experiments are representative of at least three biologically independent experiments in technical triplicate. All error bars represent mean ± SEM. Statistical tests were performed using GraphPad Prism v.6: ^∗^p < 0.05, ^∗∗^p < 0.01, ^∗∗∗^p < 0.001, and ^∗∗∗∗^p < 0.0001.

## Supplemental Information

Document S1. Supplemental Experimental Procedures, Figures S1–S4, and Tables S1 and S2

Document S2. Article plus Supplemental Information
