# Supplementary material for: Oncogenic IDH1 Mutations Promote Enhanced Proline Synthesis through PYCR1 to Support the Maintenance of Mitochondrial Redox Homeostasis
Source: Cell Rep. 2018 Mar 20;22(12):3107–14. doi: 10.1016/j.celrep.2018.02.084 (PMC5883319; doi:10.1016/j.celrep.2018.02.084)
Supplement: Document S1. Supplemental Experimental Procedures, Figures S1–S4, and Tables S1 and S2 [file mmc1.pdf]

Cell Reports, Volume *22*

Supplemental Information

Oncogenic IDH1 Mutations Promote Enhanced Proline Synthesis through PYCR1 to Support the Maintenance of Mitochondrial Redox Homeostasis

Kate E.R. Hollinshead, Haydn Munford, Katherine L. Eales, Chiara Bardella, Chunjie Li, Cristina Escribano-Gonzalez, Alpesh Thakker, Yannic Nonnenmacher, Katarina Kluckova, Mark Jeeves, Robert Murren, Federica Cuozzo, Dan Ye, Giulio Laurenti, Wei Zhu, Karsten Hiller, David J. Hodson, Wei Hua, Ian P. Tomlinson, Christian Ludwig, Ying Mao, and Daniel A. Tennant

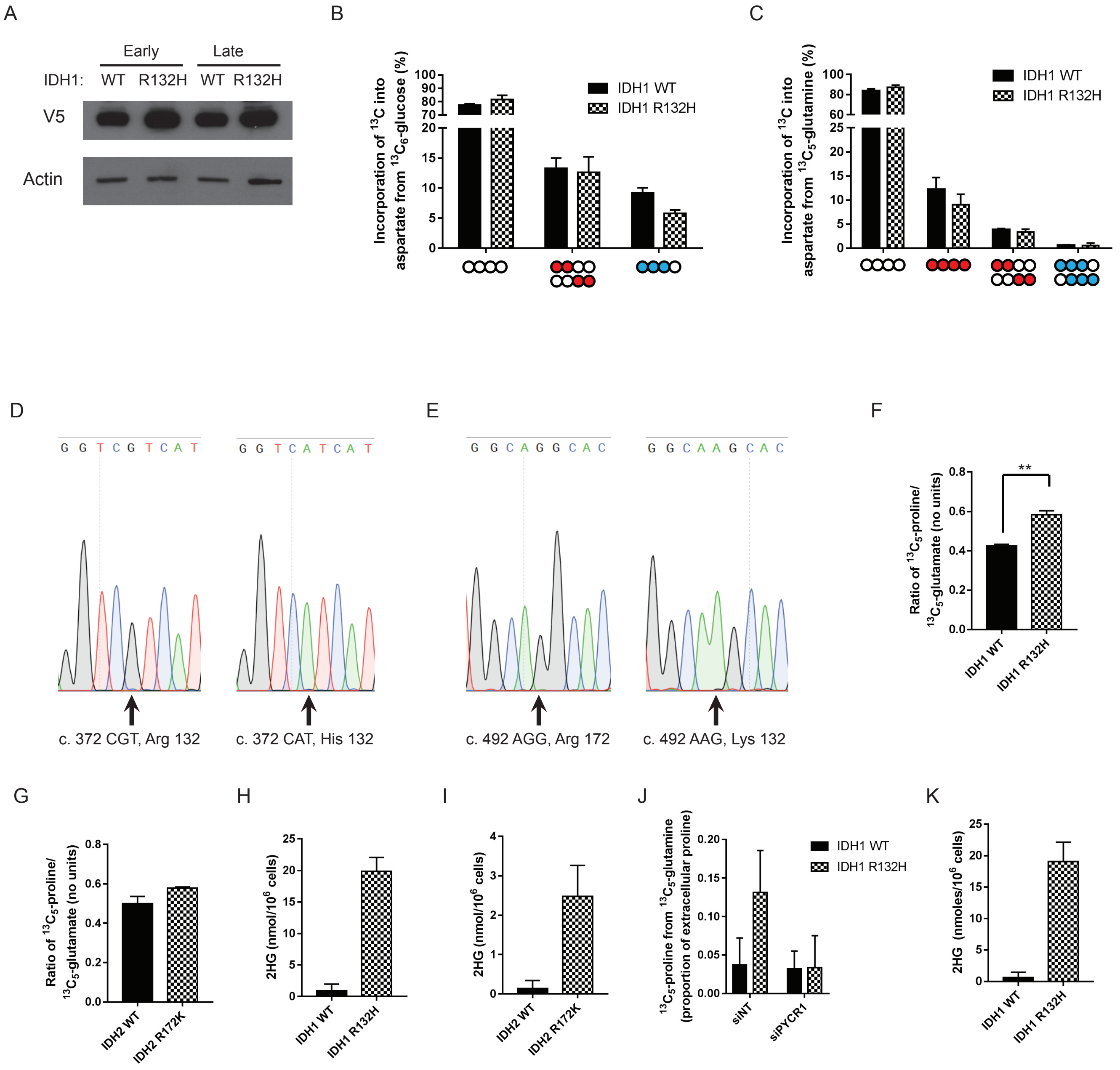

Figure S1A, Related to Figure 1. Expression of the V5-tagged IDH1 WT and R132H constructs is similar between the paired lines, and remains constant with continuous culture. **B:** Incorporation of ^{13}C from $^{13}C_6$-glucose into aspartate through oxidative pathways (red) is unchanged between IDH1 WT and IDH1 R132H-expressing cells. **C:** ^{13}C incorporation from $^{13}C_5$-glutamine into aspartate is unchanged between IDH1 WT and R132H-expressing cells. **D:** Sequencing results confirming successful transduction of R132H-mutated IDH1 (right) into LN18 parental cell lines (left). **E:** As for (**D**), but IDH2 R172K. Synthesis of proline from glutamate is increased in an IDH1 R132H-expressing (**F**), but not IDH2 R172K-expressing (**G**) LN18 cell line. Expression of IDH1 R132H (H) or R172K (**I**) in the LN18 parental cell line results in increased extracellular 2HG levels. **J:** IDH1 R132H-expressing HOG cells demonstrate increased PYCR1-mediated proline synthesis and excretion in the presence of exogenous 200 µM proline. Data presented show extracellular $^{13}C_5$-proline as a proportion of total extracellular proline. **K:** 2HG excreted into the medium in early passage IDH1 R132H-expressing HOG cell line is around 20-fold greater than in IDH1 WT-expressing controls.

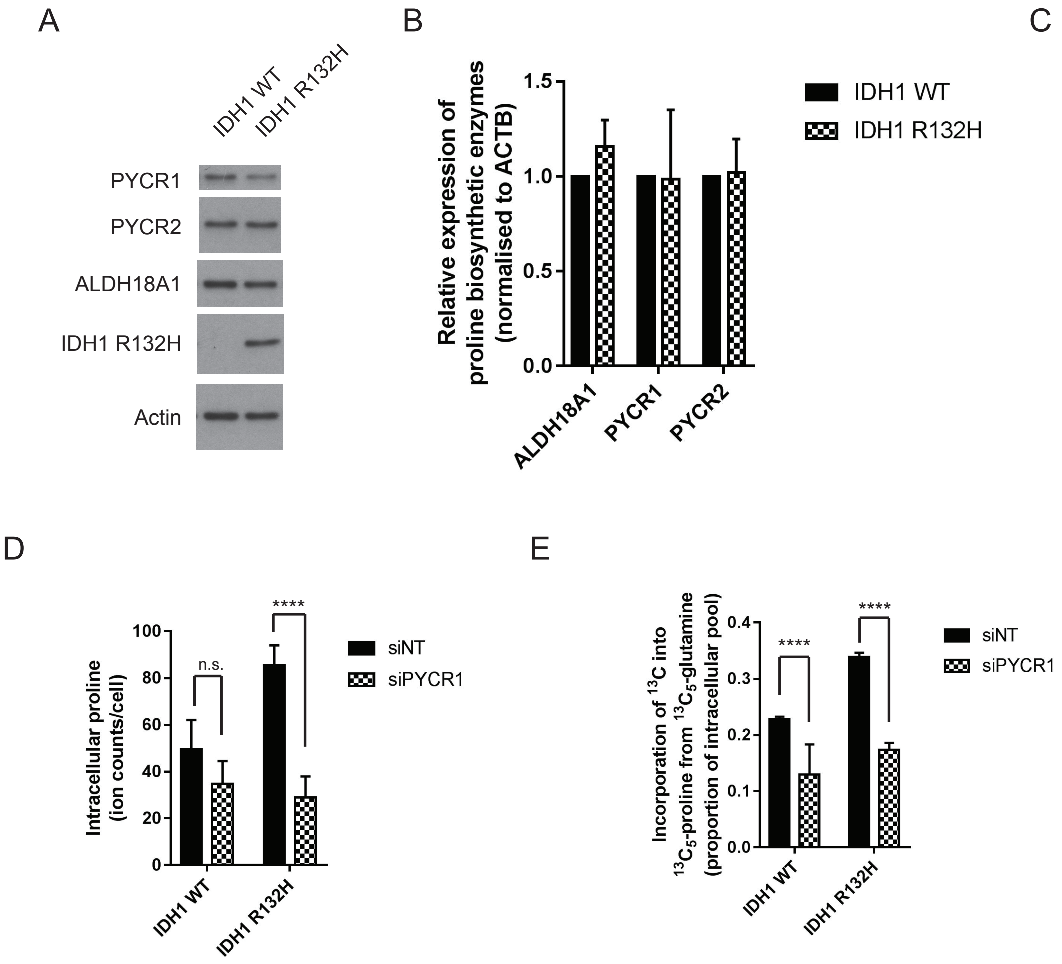

Figure S2A, Related to Figure 2: LN18 IDH1 R132H-expressing cells do not demonstrate increased expression in proline biosynthetic enzymes. **B:** Relative mRNA expression of the three mitochondrial proline biosynthetic enzymes, ALDH18A1 (pyrroline 5-carboxylate synthetase), PYCR1 and PYCR2 shows that there is no difference between the IDH1 WT and IDH1 R132H-expressing HOG cells. **C:** Immunoblot showing successful knockdown of PYCR1 in LN18 cells. **D:** LN18 cells expressing IDH1 R132H synthesize increased proline from glutamine (**E**) through PYCR1. '****' indicates p<0.0001, using a 2-way ANOVA with Sidak's multiple comparisons test.

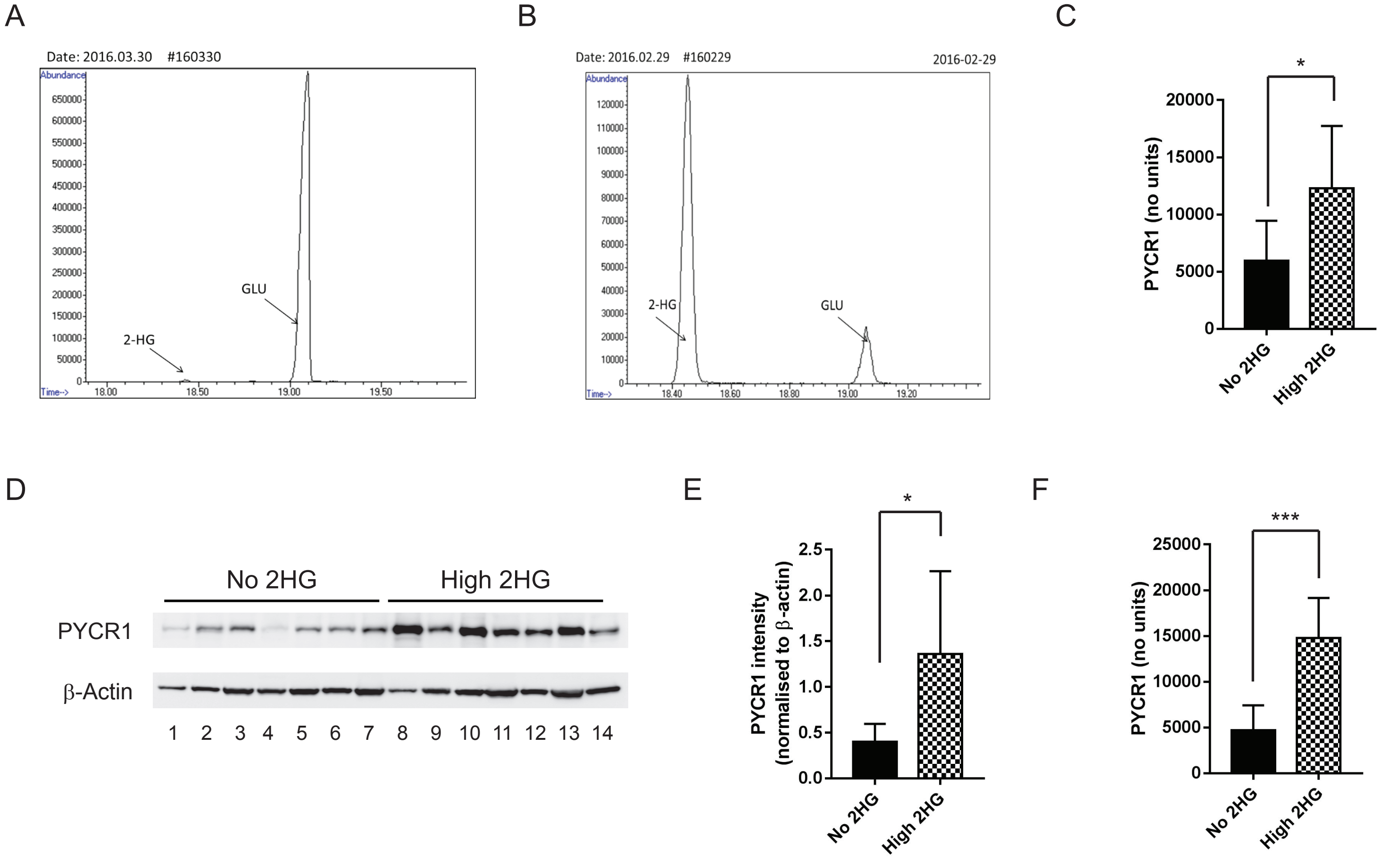

Figure S3A and B, Related to Figure 3: Representative spectra from GC-MS analysis of gliomas showing a 'no 2HG' (**A**) and high 2HG (**B**) sample. The ion at m/z 433 was used, with glutamate retention used as a control. **C:** PYCR1 densitometry normalised to protein loaded. **D:** Additional glioma samples blotted for PYCR1 (Figure 3C), separated by their tumoral 2HG levels, showing increased expression of PYCR1 both normalised to actin (**E**) and normalised to total protein loaded (**F**).

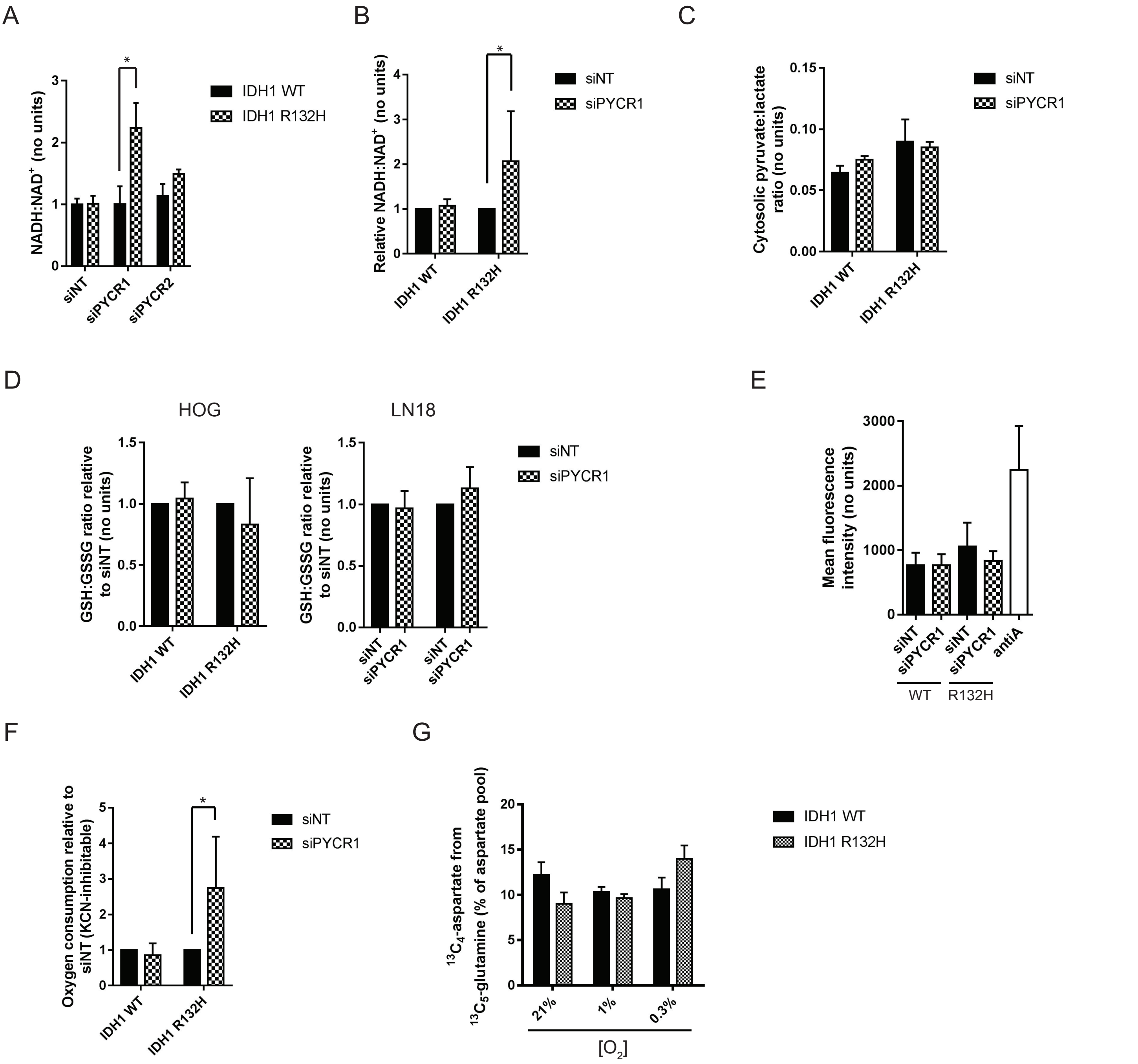

Figure S4A and B, Related to Figure 4: Knockdown of PYCR1 alters the NAD^+:NADH ratio in IDH1 R132H-expressing HOG (**A**) and LN18 (**B**) cells. No change is observed in IDH1 WT cells. '*' $p<0.05$, 2-way ANOVA with post-test. **C:** In common with the R132H-expressing HOG cells, cytosolic pyruvate:lactate ratio is unchanged after siPYCR1 in LN18 cell line. **D:** PYCR1 knockdown does not significantly alter the GSH:GSSG ratio in either wild-type or IDH1 R132H-expressing HOG (left) or LN18 (right) cells. **E:** siPYCR1 does not alter mitochondrial ROS production as measured using mitoSOX. AntiA, antimycin A positive control treatment of IDH1 WT cells. **F:** Knockdown of PYCR1 in R132H-expressing LN18 cells results in increased respiration. **G:** Oxidative aspartate synthesis is maintained in IDH1 R132H-expressing cells in limiting oxygen tensions compared with IDH1 WT cells.

Table S1

Lane # on Figure 3C	Age	Gender	Diagnosis	Sequencing	IHC for IDH1(H09)
1	62	M	GBM	Wild Type	(−)
2	64	M	GBM	Wild Type	(−)
3	46	M	AA	Wild Type	(−)
4	44	M	GBM	Wild Type	(−)
5	39	F	Oligodendroglioma	Wild Type	(−)
6	41	F	GBM	Wild Type	(−)
7	52	M	GBM	Wild Type	(−)
8	27	M	Astrocytoma	IDH1 R132H	(+)
9	50	M	Oligoastrocytoma	IDH1 R132H	(+)
10	32	F	Astrocytoma	IDH1 R132H	(+/−)
11	27	F	Astrocytoma	IDH1 R132H	(−)
12	39	F	Astrocytoma	IDH1 R132H	(+)
13	50	M	AA	IDH1 R132H	(+/−)
14	31	F	GBM	IDH1 R132H	(+)

Table S1, Related to Figure 3C: Patient characteristics for samples used in Figure 3C. Abbreviations: AA; Anaplastic astrocytoma, AO; Anaplastic oligodendroglioma, AOA; Anaplastic oligoastrocytoma, GBM; Glioblastoma.

Table S2

Diagnosis	Age	Gender	2-HG AUC	Proline AUC
AA	50	M	1220042	1337921
AO	50	M	1261259	378305
GBM	56	F	47727954	20927301
Astrocytoma	27	F	1721976	3137530
Astrocytoma	32	F	31887460	45294696
GBM	61	M	20320432	25663090
Astrocytoma	27	M	28661710	45244078
Astrocytoma	39	F	4356405	1093566
AA	30	M	39642469	29777748
AO	53	F	72633527	84155405
Oligodendroglioma	43	M	22556824	58044269
GBM	31	F	137027818	86226588
GBM	31	F	33629589	4564017
Astrocytoma	29	M	125998378	34281034
OA	50	M	97829567	61572655
OA	34	M	14875269	47941656

Table S2, Related to Figure 3E: Patient characteristics for samples used in GC-MS analysis of metabolites shown in Figure 3E. Abbreviations: AA; Anaplastic astrocytoma, AO; Anaplastic oligodendroglioma, AOA; Anaplastic oligoastrocytoma, AUC; area under the curve, GBM; Glioblastoma.

Supplemental Experimental Procedures

All chemicals were from Sigma (UK) unless otherwise specified**.**

Molecular cloning and cell transduction with lentiviral vectors

Human IDH2 cDNA was amplified by PCR using primers, which added 1X-FLAG tag to the 3' end of the sequence. This was cloned as ClaI-NheI fragment into the pCC.sin.36.MCS.PPTWpre.CMV.tTA-S2tet lentiviral transfer vector. Subsequently a recombinant PCR-based approach, using IDH2 cDNA as a template was applied to construct the IDH2 R172K mutant, which was cloned into the same lentiviral transfer vector as a ClaI-Nhe fragment, by substituting the IDH2 cassette with the IDH2-R172K mutant cassette. As a control, cells were transduced with a pCC.sin.36.eGFP.PPT.Wpre.CMV.tTA-s2tet, encoding eGFP and pCC.sin.36.IDH2WT.PPTWpre.CMV.tTA-S2tet, encoding the wild type sequence of human IDH2. PCR primers for the above are available upon request. Human IDH1 wild type and R132H mutant vectors, vector stocks and titration were prepared as described in (Bardella et al., 2016).

Cell culture

ON_TARGETplus pools used were: (L-012349-00 [PYCR1], L-016646-00 [PYCR2] or D-001810-10 [non-targeting]; Thermo Fisher, UK) at 25 nM using DharmaFECT 1 transfection reagent (Thermo Fisher, UK) 24 h post seeding. Cells were lysed at various time points post-transfection (see Figures for details) to evaluate knockdown.

Quantitative real-time PCR

Total RNA was extracted using the RNeasy Mini Kit (Qiagen, 74104) according to the manufacture's protocol. 1 µg RNA per sample was subjected to reverse transcription using Moloney Murine Leukemia Virus Reverse Transcriptase (MMLV-RT) kit (Promega, M1701). 10 µL of the resulting cDNA was used with TaqMan® gene expression master mix (AB, 4369016) for quantitative real-time PCR using AB 7500 Real Time PCR System. The

following primers and probes were used: *PYCR1* (Hs01048016_m1), *PYCR2* (Hs01016460_gH), *ALDH18A1* (Hs00913261_m1), *ACTIN* (Hs01060665_g1) (ThermoFisher Scientific). The expression of ALDH18A1, PYCR1 and PYCR2 were normalised to actin as housekeeping gene. Comparative analysis across samples was calculated using the $2^{-\Delta\Delta CT}$ method.

O_2 Consumption Measurements

Cells were seeded at 2 x 10^5 onto 6-well plates in standard culture conditions and were transfected 24 h later with targeting siRNA against PYCR1 and PYCR2. The cells were harvested and re-suspended in complete media. 4 x 10^5 cells in 300 µl were added to the electrode chamber and the oxygen consumption rate was measured over a 5 min period using Oxygraph software (http://www.hansatech-instruments.com/). Cells were kept in suspension using a stirring bar and the chamber temperature was maintained at 37°C through the use of a heating block. After recording for 5 min, KCN 700 µM (Sigma-Aldrich, 60178) was added to the electrode chamber to inhibit cellular respiration and the oxygen consumption was measured for a further 3 min. The final respiration rate was obtained by subtracting the oxygen consumption rate in presence of KCN to the oxygen consumption rate in the absence of KCN.

NMR Spectroscopy

4 x 10^6 cells were plated onto 15 cm dishes and cultured in standard medium overnight. Media was replaced with basic formulation DMEM supplemented with $^{13}C_6$ glucose or $^{13}C_5$ glutamine at 10 mM and 2 mM respectively for 24 h prior to extraction. At the conclusion of tracer experiments, cells were washed with ice-cold 0.9% saline solution and extracted in 1.2 mL pre-chilled methanol (-20°C), water (4°C) and chloroform (-20°C) in a 1:1:1 ratio. Cell lysates were vortexed for 15 min at 4°C and immediately centrifuged at 15,000 *g* for 15 min at 4°C. Extraction was performed on three different cultures for each labelling experiment.

Dried samples were re-suspended in 60 µL of 100 mM sodium phosphate buffer (pH7.0) containing 500 µM DSS and 2 mM Imidazole, 10% D_20, pH 7.0. Samples were vortexed, sonicated (5-15 min) and centrifuged briefly, before transferred to 1.7 mm NMR tubes using an automated Gilson.One-dimensional (1D)-^{1}H-NMR spectra and two-dimensional (2D)-^{1}H,^{13}C-Heteronuclear Single Quantum Coherence Spectroscopy (HSQC) NMR spectra were acquired using a 600-MHz Bruker Avance III spectrometer (Bruker Biospin) with a TCI 1.7 mm z-PFG cryogenic probe at 300 K. Spectral widths were set to 7,812.5 and 24,155 Hz for the ^{1}H and ^{13}C dimensions, respectively. 16,384 complex data points were acquired for the 1D-spectra and 512 complex data points were acquired for the ^{1}H dimension of 2D-^{1}H,^{13}C-HSQC NMR spectra. An exponentially weighted non-uniform sampling scheme was used for the indirect dimension. Here, 30% of 8,192 complex data points (2,458) were acquired. 128 transients were recorded for the 1D-NMR spectra with a relaxation delay of 4 s, and two transients were recorded for the 2D-^{1}H,^{13}C-HSQC NMR spectra with a relaxation delay of 1.5 s. Each sample was automatically tuned, matched and then shimmed (1D-TopShim) to a DSS line width of <2 Hz before acquisition of the first spectrum. Total experiment time was ~15 min per sample for 1D-^{1}H-NMR spectra and 4.5 h per sample for 2D-^{1}H,^{13}C-HSQC NMR spectra. 1D-^{1}H-NMR spectra were processed using the MATLAB-based MetaboLab software (Ludwig and Gunther, 2011). All 1D data sets were zero-filled to 131,072 data points before Fourier Transformation. The chemical shift was calibrated by referencing the DSS signal to 0 p.p.m. 1D-spectra were manually phase corrected. Baseline correction was achieved using a spline function (Ludwig and Gunther, 2011). 1D-^{1}H-NMR spectra were exported into Bruker format for metabolite identification and concentration determination using Chenomx 7.0 (ChenomxINC). 2D-^{1}H,^{13}C-HSQC NMR spectra were reconstructed using compressed sensing in the MDDNMR and NMRpipe software (Delaglio et al., 1995; Kazimierczuk and Orekhov, 2011; Orekhov and Jaravine, 2011). The final spectrum size was 1,024 real data points for the ^{1}H dimension and 16,384 real data points for the ^{13}C dimension. Analysis was performed using MetaboLab and pyGamma software was used in multiplet simulations (Smith et al., 1994). The methyl group of lactate was used

to calibrate the chemical shift based on its assignment in the human metabolome database (Wishart et al., 2013).

GC-MS

Cell analysis - cells were seeded at 2 x 10^5 onto 6-well plates in standard culture conditions and transfected with non-targeting RNA (siNT) and siRNA targeting PYCR1 (siPYCR1) and PYCR2 (siPYCR2) at 25 nM. Media was changed to basic formulation DMEM containing either 10 mM $^{13}C_6$ glucose or 2 mM $^{13}C_5$ glutamine with the other carbon source unlabelled, for 24 h prior to extraction. At the conclusion of tracer experiments, cells were washed with 2 mL ice-cold 0.9% saline solution and quenched with 0.3 mL pre-chilled methanol (-20°C). After adding an equal volume of ice-cold HPLC-grade water containing 1 µg/mL D6-glutaric acid (C/D/N Isotopes Inc), cells were collected with a cell scraper and transferred to tubes containing 0.3 mL of chloroform (-20°C). The extracts were shaken at 1400 rpm for 20 min at 4°C and centrifuged at 16,000 *g* for 5 min at 4°C. 0.3 mL of the upper aqueous phase was collected and evaporated in GC glass vials under vacuum at -4°C using a refrigerated CentriVap Concentrator (Labconco). Metabolite derivatization was performed using an Agilent autosampler. Dried polar metabolites were dissolved in 15 µl of 2% methoxyamine hydrochloride in pyridine (Thermo Fisher Scientific, 25104) at 55°C, followed by an equal volume of N-tert-Butyldimethylsilyl-N-methyltrifluoroacetamide with 1% tert-butyldimethylchlorosilane after 60 minutes, and incubation for a further 90 min at 55°C.

GC-MS analysis was performed using an Agilent 6890GC equipped with a 30 m DB-35MS capillary column. The GC was connected to an Agilent 5975C MS operating under electron impact ionization at 70 eV. The MS source was held at 230°C and the quadrupole at 150°C. The detector was operated in scan mode and 1 µL of derivatised sample was injected in splitless mode. Helium was used as a carrier gas at a flow rate of 1 mL/min. The GC oven temperature was held at 80°C for 6 min and increased to 325°C at a rate of 10°C/min for 4

min. The run time for each sample was 59 min. Measurements in selcted ion monitoring (SIM) mode were performed as described previously (Battello et al., 2016). For determination of the mass isotopomer distributions (MIDs), spectra were corrected for natural isotope abundance. Data processing from raw spectra to MID correction and determination was performed using MetaboliteDetector software (Hiller et al., 2009).

Tumor metabolite analysis

20 mg of tumor was mixed with 400 µL of 80% methanol, homogenized in a Tissuelyser-48 (Shanghai Jingxin), and then centrifuged at 14,000 rpm for 10 min at 4°C. A 200 µL aliquot of supernatant was transferred to a screw-cap V-type glass-bottom vial and dried in a vacuum-drying apparatus at 30°C for 1 h. Methoxyamine hydrochloride was dissolved in pyridine at a concentration of 20 mg/mL; 35 µL of this solution was added to the sample, and then incubated at 70°C for 0.5 h. Derivatization was performed at 70°C for 40 min following addition of 20 µL of MTBSTFA. The sample (1 µL) was subjected to GC-MS, with a total run time of 32 min. A capillary column (HP-5ms Intuvo, 30 m × 0.25 mm × 0.25 µm; Agilent Technologies) using helium as a carrier gas was utilized for GC separation. The inlet model was splitless, and its temperature was 250°C. Parameters for GC-MS were as follows: 100°C for 3 min; ramp 10°C/min to 140°C, 8°C/min to 260°C, and 10°C/min to 310 °C; and then hold for 5 min at 310°C. The total run time was 32 min. The temperature of the injector was set at 280°C, and that of the MSD Transfer Line was 290°C. Ion source was EI (70 eV) at a temperature of 230°C. Mass scan range was from 50 to 600 m/z.

Redox measurements

Cells were seeded at 2 x 10^4 onto 8-well chamber slides (Thistle Scientific, IB-80826) for NAD(P)H autofluorescence measurements, or 6 x 10^3 in 96-well plates for NAD:NADH

assay. Both were transfected 24 h later with targeting and non-targeting siRNA against PYCR1 or PYCR2 where shown before NAD(P)H imaging or biochemical analysis 48 h later. NAD(P)H was excited at λ = 351/364 nm using the argon UV laser module on a Zeiss UV Axiovert confocal autofluorescence, and autofluorescence captured at λ = 385-470 nm using a photomultiplier tube (PMT) (1024 x 1024 pixels; 12-bit). Carbonyl cyanine m-chlorophenyl hydrazine (CCCP) 20 µM (Sigma-Aldrich, C2759) and Rotenone 60 µM (Sigma-Aldrich, R8875) were added to each well to achieve basal and maximal NAD(P)H autofluorescence, respectively. User-blinded offline analysis was performed using Fiji software (http://fiji.sc/) and 48-126 random visual fields were analyzed per experimental condition. Images presented were exported into ImageJ, and noise reduction performed using the 'remove outliers' function with standard settings. NAD:NADH assay (NAD/NADH Glo™ Assay (Promega, G9071) was performed as per manufacturer's protocol.

ROS measurements

Cells were seeded at 2 x 10^5 onto 6-well plates in standard culture conditions and as described above. To assay for ROS, media was replaced with HBSS before incubation with 5 µM of MitoSOX Red (Thermofisher, M36008) at 37°C for 10 minutes. The cells were trypsinized and subsequently collected in HBSS with 1% FBS. Using an LSRFortessa X-20 flow cytometer, fluorescence at 575nm was measured and mean fluorescence intensity of 10,000 events in triplicate assessed using FloJo software.

SRB assay

Cells were treated with 50 nM rotenone for 72 h prior to being fixed in 20% (v/v) ice-cold trichloroacetic acid solution (TCA) (Sigma-Aldrich, T0699) for 30 min at 4°C. Plate wells were washed with water and once dry, intracellular protein was stained using 0.4% (w/v)

sulfohodamine B (SRB) (Sigma-Aldrich, 230162) in 1% acetic acid for 10 min at room temperature. After washing with 1% acetic acid to reduce non-specific staining, SRB was dissolved in 50 mM Tris/HCl pH 8.8 once dry. 100 µL/well was aliquoted for quantification by absorbance at 495 nm on FLUOstar Omega (BMG LabTech). Final sample absorbance values were determined by calculating the mean blank-corrected absorbance for each replicate, where 50 mM Tris/HCl pH 8.8 alone was used as the blank.

References

Bardella, C., Al-Dalahmah, O., Krell, D., Brazauskas, P., Al-Qahtani, K., Tomkova, M., Adam, J., Serres, S., Lockstone, H., Freeman-Mills, L., *et al.* (2016). Expression of Idh1(R132H) in the Murine Subventricular Zone Stem Cell Niche Recapitulates Features of Early Gliomagenesis. Cancer Cell *30*, 578-594.

Battello, N., Zimmer, A.D., Goebel, C., Dong, X., Behrmann, I., Haan, C., Hiller, K., and Wegner, A. (2016). The role of HIF-1 in oncostatin M-dependent metabolic reprogramming of hepatic cells. Cancer Metab *4*, 3.

Delaglio, F., Grzesiek, S., Vuister, G.W., Zhu, G., Pfeifer, J., and Bax, A. (1995). NMRPipe: a multidimensional spectral processing system based on UNIX pipes. J Biomol NMR *6*, 277-293.

Hiller, K., Hangebrauk, J., Jager, C., Spura, J., Schreiber, K., and Schomburg, D. (2009). MetaboliteDetector: comprehensive analysis tool for targeted and nontargeted GC/MS based metabolome analysis. Anal Chem *81*, 3429-3439.

Kazimierczuk, K., and Orekhov, V.Y. (2011). Accelerated NMR spectroscopy by using compressed sensing. Angew Chem Int Ed Engl *50*, 5556-5559.

Ludwig, C., and Gunther, U.L. (2011). MetaboLab--advanced NMR data processing and analysis for metabolomics. BMC Bioinformatics *12*, 366.

Orekhov, V.Y., and Jaravine, V.A. (2011). Analysis of non-uniformly sampled spectra with multi-dimensional decomposition. Prog Nucl Magn Reson Spectrosc *59*, 271-292.

Smith, S.A., Levante, T.O., Meier, B.H., and Ernst, R.R. (1994). Computer-Simulations in Magnetic-Resonance - an Object-Oriented Programming Approach. J Magn Reson Ser A *106*, 75-105.

Wishart, D.S., Jewison, T., Guo, A.C., Wilson, M., Knox, C., Liu, Y., Djoumbou, Y., Mandal, R., Aziat, F., Dong, E., *et al.* (2013). HMDB 3.0--The Human Metabolome Database in 2013. Nucleic Acids Res *41*, D801-807.
